# A Suggestion for the Establishment of a Physicians’ Club

**Published:** 1903-03

**Authors:** Benjamin H. Grove

**Affiliations:** 334 Pearl Street


### CORRESPONDENCE.

### A Suggestion for the Establishment of a Physicians' Club.

*Editor Buffalo Medical Journal :*

SIR: The spirit of *camaraderie* is none too well developed in the medical profession. Much of this condition would undergo a change for the better if the opportunities for social meetings of physicians were more frequent. At a recent meeting of the Medical Society of the County of Erie a member of the legal profession, who participated in the discussion, voiced his opinion of some of the shortcomings of the members of the medical profession. As physicians we need, I think, to get more in touch with each other and thus would we learn to know one another better and develop a more becoming fellowship. How can this be brought about?

During the enjoyable reception given by Dr. Crego, February 4, in honor of Dr. Hall, the undersigned, from more than a score of gentlemen present, received a hearty response to the suggestion of the formation of a physicians' club. Such clubs exist in other cities and Philadelphia boasts of a very flourishing one of over 500 members. A number of good reasons might be presented for the establishment of such a club among the physicians of Buffalo, but this communication is merely a suggestion. Many reputable physicians are not eligible for membership in the University Club, on account of its exclusive requirements—the degree of A. B., or its equivalent being necessary. For many, the other prominent clubs are too expensive. What say you? If you favor the formation of such a club, please communicate with Dr. William Warren Potter, the editor of this JOURNAL, or the undersigned.

BENJAMIN H. GROVE.

334 PEARL STREET, February 17, 1903.

---

H. CAMPBELL THOMSON corroborates Lauder Brunton's observations (*British Med. Jour.*) on the use of alkalies for the relief of pain in neuralgia and other peripheral nerve lesions. In a severe and obstinate neuralgia great relief was experienced from an alkaline mixture of which ammonium carbonate was the principal ingredient.

